# Dynamic Condition Adversarial Adaptation for Fault Diagnosis of Wind Turbine Gearbox

**DOI:** 10.3390/s23239368

**Published:** 2023-11-23

**Authors:** Hongpeng Zhang, Xinran Wang, Cunyou Zhang, Wei Li, Jizhe Wang, Guobin Li, Chenzhao Bai

**Affiliations:** Marine Engineering College, Dalian Maritime University, No. 1 Linghai Road, Dalian 116000, Chinaw1120211123@dlmu.edu.cn (X.W.); dmuliwei@dlmu.edu.cn (W.L.); wangjizhe@dlmu.edu.cn (J.W.); liguobin@dlmu.edu.cn (G.L.)

**Keywords:** wind turbine, fault diagnosis, adversarial domain adaptation, semi-supervised learning

## Abstract

While deep learning has found widespread utility in gearbox fault diagnosis, its direct application to wind turbine gearboxes encounters significant hurdles. Disparities in data distribution across a spectrum of operating conditions for wind turbines result in a marked decrease in diagnostic accuracy. In response, this study introduces a tailored dynamic conditional adversarial domain adaptation model for fault diagnosis in wind turbine gearboxes amidst cross-condition scenarios. The model adeptly adjusts the importance of aligning marginal and conditional distributions using distance metric factors. Information entropy parameters are also incorporated to assess individual sample transferability, prioritizing highly transferable samples during domain alignment. The amalgamation of these dynamic factors empowers the approach to maintain stability across varied data distributions. Comprehensive experiments on both gear and bearing data validate the method’s efficacy in cross-condition fault diagnosis. Comparative outcomes demonstrate that, when contrasted with four advanced transfer learning techniques, the dynamic conditional adversarial domain adaptation model attains superior accuracy and stability in multi-transfer tasks, making it notably suitable for diagnosing wind turbine gearbox faults.

## 1. Introduction

As an essential component in the wind turbine’s transmission system, the gearbox often operates under variable loads and is prone to failures [1]. Absolutely, conducting fault diagnosis on the gearbox of wind turbines (WTs) is highly necessary. The conventional fault diagnosis techniques relying on vibration signals demand substantial prior knowledge and expert experience [2]. With the advancement of artificial intelligence technology, rotating machinery fault diagnosis methods based on deep learning such as NN, LSTM, and DBN have experienced rapid development [3]. These methods are also applied to the fault diagnosis of the WT gearbox [4,5,6,7,8,9]. Huang et al. utilized wavelet packet decomposition to process the vibration signals of the gearbox. They then used the decomposed signals as inputs to a multi-scale convolutional neural network, enabling intelligent fault diagnosis of WT gearbox. The effectiveness of this method was validated through real operational data from WTs [10]. However, all these approaches assume that the distribution of the training and testing data is identical. Indeed, WTs often operate under variable working conditions, and the data distribution varies across different conditions [11]. As a result, the model’s accuracy can significantly decrease when faced with different operating conditions [12,13]. Additionally, labeling a large amount of data for all operating conditions comes with a high cost. This limitation hinders the practical application of deep learning in WT fault diagnosis.

In order to tackle the aforementioned challenges, domain adaptation methods are applied in cross-domain fault diagnosis [14]. Domain adaptation is achieved by employing a novel optimization strategy to align the feature distribution of target domain data with the source domain, thereby reducing the differences in feature distribution between domains. When the target domain data lack sufficient labels, employing a semi-supervised learning strategy. This approach involves extracting transferable features from the labeled data in the source domain and utilizing them to train a model capable of accurately classifying data in the target domain. In the field of domain adaptation, some researchers utilize distance metrics to align the marginal distributions of the source and target domains. Simultaneously, they integrate deep neural networks to achieve cross-domain fault classification. By aligning the marginal distributions, the model can effectively reduce the distribution discrepancy between the domains, facilitating accurate fault detection and classification across different domains, even in scenarios with limited or no labeled data in the target domain. This approach enables the model to transfer knowledge learned from the source domain and generalize it to the target domain, improving the overall performance and adaptability of the fault classification system [15,16,17,18,19]. Lu et al. achieved cross-domain fault diagnosis by combining the maximum mean discrepancy (MMD) metric with deep neural networks. This approach enabled them to align the distributions of data between the source and target domains effectively. By minimizing the MMD, the model reduces the domain discrepancy, allowing the deep neural network to learn transferable features from the source domain and generalize them to the target domain [20]. Zhu et al. proposed a transfer learning algorithm based on an improved Wasserstein distance to achieve cross-condition fault diagnosis for bearings. This algorithm is capable of extracting domain-invariant features, thereby reducing the impact of domain shift on the model’s classification accuracy.

By utilizing the improved Wasserstein distance, the algorithm effectively measures the dissimilarity between the source and target domains. This distance metric allows the model to align the distributions of data from different working conditions, mitigating the domain shift problem. Consequently, the model can identify and extract features that are relevant across various conditions, enhancing its ability to accurately diagnose faults in bearings even under different operational scenarios. The use of domain-invariant features ensures that the model remains robust and consistent across different working conditions, ultimately leading to improved fault diagnosis performance [21]. Han et al. drew inspiration from Generative Adversarial Networks (GANs) and proposed a Deep Adversarial Convolutional Neural Network (DACNN) model to address cross-domain fault diagnosis in WT gearbox systems. The model comprises three main components: a feature extractor, a domain discriminator, and a classifier. It utilizes adversarial learning between the domain discriminator and feature extractor to reduce the distribution gap between domains. Through the utilization of the domain discriminator, the model acquires the ability to differentiate features originating from distinct domains, while the feature extractor strives to produce representations that are invariant across domains. Through this adversarial training process, the model effectively aligns the feature distributions across different domains, facilitating the transfer of knowledge from the source domain to the target domain. The model’s effectiveness was demonstrated through validation on wind power generation testbeds and industrial gearbox datasets [22].

The above domain adaptation algorithms only focus on reducing the distance between the marginal distributions of the source and target domains. However, in complex classification tasks, even if the marginal distributions are successfully aligned, it does not guarantee good classification performance [23]. Therefore, some researchers adopt a method that optimizes the inter-class distance for domain alignment, aiming to improve the model’s generalization ability in multi-class problems. The key to this approach is to optimize the distance between different classes, making the same categories from the source and target domains closer to each other and dissimilar categories farther apart. The model can better learn shared features and category structures between the source and target domains, thereby enhancing its generalization ability in multi-class problems. This class-level distance-based domain alignment method can achieve better performance in more complex real-world scenarios and provides an effective solution for cross-domain classification tasks [24,25,26,27,28,29]. Chen et al. proposed a multi-kernel domain adaptive network for fault diagnosis in WT systems. This model utilizes multiple kernels to align each sub-class separately by minimizing the maximum mean discrepancy (MMD) in different feature spaces. Experimental results demonstrate its stable performance in scenarios with speed fluctuations. However, due to the requirement of simultaneously optimizing multiple objective functions, the training speed of the model is significantly reduced [30]. Xia et al. built upon domain adversarial networks and proposed a method that uses the conditional distribution between feature vectors generated by the feature extractor and pseudo-labels generated by the classifier as the input for the domain discriminator. They introduced an adaptive weighting mechanism based on mean squared error (MSE) to balance the learning between the classifier and domain discriminator. The effectiveness of this approach was validated on an industrial robot joint-bearing dataset [31]. An et al. proposed a domain adaptive network based on contrastive learning for cross-condition bearing fault diagnosis. This model aligns the marginal distributions by minimizing the maximum mean discrepancy and simultaneously aligns the sub-domains using a conditional domain adversarial approach. This approach allows the model to learn domain-invariant features that are relevant to fault diagnosis across different working conditions. The effectiveness of this method has been demonstrated through experiments, making it a promising approach for cross-condition fault diagnosis in practical applications [32]. Due to the WT gearbox often operating in variable work environments, the introduction of domain adaptation methods becomes essential for achieving high-accuracy fault diagnosis when only single-working-condition labeled data are available. Moreover, given the significant differences between various working conditions, traditional domain adaptation methods fail to effectively integrate the alignment of marginal and conditional distributions. During the training process, ensuring the balance between these two is also crucial, aiding the model to adapt to more complex working conditions.

Based on this, this paper proposes a semi-supervised fault diagnosis method for WT gearbox systems based on Dynamic Conditional Adversarial Adaptation (DCAA). The main contributions of this paper are summarized as follows:

1. The DCAA method jointly adjusts the marginal and conditional distributions between two different domains. By introducing dynamic weighting factors, it dynamically measures the relative importance of marginal and conditional alignment, enhancing the accuracy of feature distribution alignment.
2. The method employs information entropy as a metric to assess the reliability of classifier predictions. It prioritizes using data with high confidence for domain adaptation training, thereby improving the fault diagnosis accuracy under complex working conditions.
3. Through a series of experiments, the effectiveness of DCAA is demonstrated in both WT gearbox and bearing fault diagnosis scenarios. Furthermore, the method’s superiority is validated by comparing it with other domain adaptation approaches.

The remaining sections are as follows. Section 2 introduces the fundamental theory of adversarial domain adaptation. Section 3 presents the proposed DCAA (Dynamic Conditional Adversarial Adaptation) model. In Section 4, comparative experiments are conducted to validate and analyze the results of the DCAA model. Finally, Section 5 provides a comprehensive summary and conclusion of the paper.

## 2. Related Work

### 2.1. Problem Description

**Figure 1:**
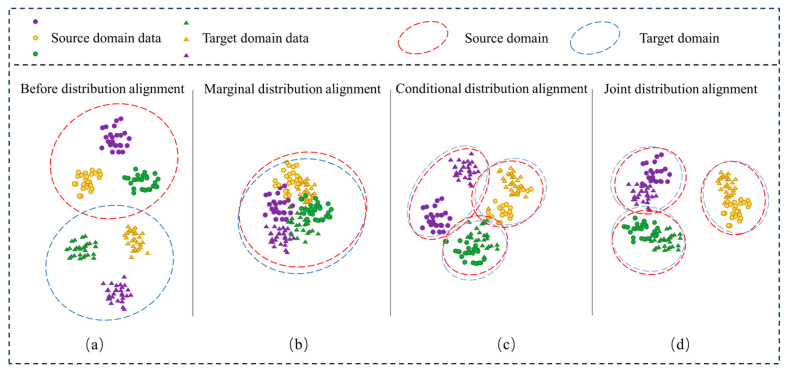
Effects of different distribution alignments. (**a**) Presents the data distribution of the source and target domains prior to distribution alignment. Meanwhile, (**b**), (**c**), and (**d**), respectively, illustrate the effects of marginal distribution alignment, conditional distribution alignment, and joint distribution alignment.

The high accuracy of intelligent fault diagnosis is contingent upon a critical premise: the training and test data must come from the same distribution. Hence, in the fault diagnosis of WT gearboxes, we are often limited to labeled data from a single working condition due to challenges in data acquisition. However, the data distribution in actual working conditions (target domain data) often differs from that of the existing conditions (source domain data) due to changes in working conditions, such as speed and load, as illustrated in Figure 1a. This discrepancy significantly reduces the model’s classification accuracy. We resolve this issue by aligning the data distributions of the source and target domains. Figure 1b–d represent the results of marginal distribution alignment, conditional distribution alignment, and joint alignment methods combined, respectively. Marginal distribution alignment can significantly reduce the distance between domains, but it may lead to confusion since it treats all data points equally. On the other hand, conducting only conditional distribution alignment can result in some classes still having large domain distances, which hinders effective classification. Therefore, in complex transfer tasks, it is crucial to simultaneously consider both domain adaptation methods and dynamically adjust them based on the data distribution to achieve the best transfer performance.

This paper investigates fault diagnosis in wind turbine gearbox systems under diverse working conditions. The problem is described as follows: This research involves collecting vibration signals from different working conditions, which serve as the source domain $D_{s}={\left\{(x_{i}^{s},y_{i}^{s})\right\}}_{i=1}^{n}$ containing *m* labeled data points, following the joint distribution $D(x^{s},y^{s})$ and the target domain $D_{s}={\left\{(x_{i}^{s},y_{i}^{s})\right\}}_{i=1}^{n}$ containing *n* unlabeled data points, following the joint distribution $Q(x^{t},y^{t})$. The vibration signals from different working conditions have distinct marginal feature distributions but share the same label space. The objective of DCAA is to design a deep neural network model y=f(x) that reduces the feature distribution distance between the two domains, such that the target risk $\in_{t}(f)=E_{(x^{t},y^{t})\sim Q}[f(x^{t})\neq y^{t}]$ can be constrained by adding the domain distance disc(P,Q) to the source risk $\in_{s}(f)=E_{(x^{s},y^{s})\sim P}[f(x^{s})\neq y^{s}]$. The domain distance disc(P,Q)*disc* (*P*, *Q*) is quantified using a domain discriminator and a conditional domain discriminator.

### 2.2. Conditional Adversarial Domain Adaptation

Conditional adversarial domain adaptation (CADA) is an advanced transfer learning method. The CADA network consists of three components: the feature extractor *F*, the classifier *G*, and the domain discriminator *D*. During forward propagation, the feature extractor F is responsible for extracting information from the data and encoding it into feature vectors. The classifier *G* uses these feature vectors to classify the data and generates pseudo-labels. The domain discriminator *D*, on the other hand, utilizes the information from the feature vectors and pseudo-labels to determine whether the data come from the source domain or the target domain.

(1) $$LossG=E_{x_{i}^{s}\sim D_{s}}\sum_{c=1}^{n}1_{\left(y_{c=j}^{s}\right)}\log[G_{c}(f_{i}^{s})]$$

where $f_{i}^{s(t)}$ represents the feature vector of the *i*-th sample from the source (or target) domain, i.e., $f_{i}^{s(t)}=F(x_{i}^{s(t)})$. $g_{i}^{s(t)}$ represents the pseudo-label of the *i*-th sample from the source (or target) domain, i.e., $g_{i}^{s(t)}=G(x_{i}^{s(t)})$. $D\left(\cdot \right)$ *D*() takes values between 0 and 1, indicating whether the input data come from the source domain or the target domain.

During the training process, the objective is to minimize the classification loss while simultaneously maximizing the domain discriminator’s loss. Adversarial training is employed to reduce the distribution discrepancy between the source and target domain features. The optimization objective can be represented as follows:

(2) $$\underset{G}{\min}LossG-LossD$$

(3) $$\underset{D}{\min}LossD$$

Through the optimization process described above, the feature extractor *F* is encouraged to extract features that are invariant to domain shifts, making it difficult for the domain discriminator *D* to distinguish the source and target domains. At the same time, the classifier *G* is fine-tuned to improve its accuracy in classifying the source domain data. This dual optimization strategy allows the model to effectively classify unlabeled data in the target domain, achieving domain adaptation and enhancing the model’s generalization to target domain samples.

## 3. Proposed Method

### 3.1. Subsection

**Figure 2:**
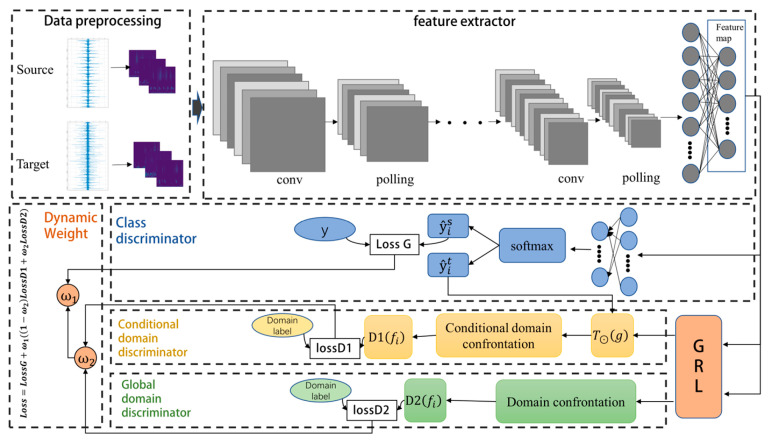
Structure of CDAA Model.

As shown in Figure 2, the DCAA model consists of feature extractor, classifier, domain discriminator, and conditional domain discriminator. The feature extractor *F* is responsible for extracting domain-invariant features from the source domain and target domain data, encoding them into feature vectors f. Classifier *C* is able to perform fault classification utilizing feature vectors. The domain discriminator *D*1 and conditional domain discriminator *D*2 are used to align the marginal distribution and conditional distribution, respectively. Additionally, dynamic weighting factors *ω*_1_ and *ω*_2_ are introduced to adjust the model training process, enhancing the model’s generalization capability and robustness.

The optimization objectives for the classifier, domain discriminator, and conditional domain discriminator are expounded in Section 3.2.1, Section 3.2.2 and Section 3.2.3, respectively. Furthermore, the methodology for defining dynamic weights and their functional role in model optimization is elucidated in Section 3.2.4.

In the subsequent case studies of this article, the architecture of the feature extractor is composed of three convolutional layers, three pooling layers, and two dropout layers, all employing the ReLU activation function. For specific model parameters, please refer to Table 1.

### 3.2. Optimization Strategy

#### 3.2.1. Classifier G

The classifier, employing the feature vector f as input, adjudicates its fault category. The output of the classifier is denoted as $\hat{y}$. We train the model using labeled data from the source domain. Specifically, this is achieved by optimizing the parameters of both the feature extractor and classifier to minimize the loss between $\hat{y}$ and $y_{s}$, which ensuring that f encompasses discriminative features. The loss function between $\hat{y}$ and $y_{s}$ can be articulated as follows:

(4) $$R_{c}(G)=E_{(x_{s},y_{s})\sim P}L(\hat{y},y_{s})$$

where: $L(\hat{y},y_{s})$ represents the cross-entropy loss function. $\hat{y}$ denotes the predictions made by classifier *G*. $y_{s}$ represents the ground truth labels.

#### 3.2.2. Domain Discriminator *D*1

Due to domain shift, a classifier that is solely trained on source domain data might not exhibit satisfactory performance when applied to target domain data. We introduce a domain discriminator to differentiate the data sources. The domain discriminator’s discrimination loss can be expressed as

(5) $$R_{d1}(G,D1)=E_{(x_{i}^{s})\sim G_{s}}\log[D1(f_{i}^{s})]+E_{(x_{j}^{t})\sim G_{s}}\log[1-D1(f_{j}^{t})]$$

where $f_{i}^{s}$ and $f_{j}^{t}$, respectively, denote the feature vectors of the source domain data and the target domain data, i.e., $f_{i}^{s},f_{j}^{t}\in f$. Domain discriminator *D*1 is a binary classification model, and its loss is calculated using a binary cross-entropy function. Therefore, the output of $D1(f_{i}^{s})$ and $D1(f_{j}^{t})$ are in the range [0, 1]. From Equation (5), it can be inferred that an increase in domain discrimination loss results in a decrease in the accuracy of domain discrimination. This indicates that the *F* is capable of extracting domain-invariant features, thereby effectively confusing the domain discriminator.

#### 3.2.3. Conditional Domain Discriminator *D*2

In multi-class tasks, relying solely on the domain discriminator *D*1 for domain alignment is insufficient. This often leads to a situation where the extracted features can successfully confuse the discriminator, but the classification performance on the target domain remains unsatisfactory. This is because the domain discriminator narrows the distance between features of different classes in the source and target domains. Therefore, it is necessary to add a conditional domain discriminator to align the classes. The conditional domain discriminator *D*2 is trained by conditioning the feature vector f on the predicted label $\hat{y}$. It utilizes the joint variable $g=(f,\hat{y})$ of $\hat{y}$ and f to train the conditional domain discriminator *D*2. The domain discriminator loss function can be expressed as

(6) $$R_{d2}(G,D2)=E_{(x_{i}^{s})\sim G_{s}}\log[D2(T(g_{i}^{s}))]+E_{(x_{j}^{t})\sim G_{s}}\log[1-D2(T(g_{j}^{t}))]$$

where $T(g)=T(f,\hat{y})$ is a random multi-thread mapping between feature vector *f* and predicted label $\hat{y}$:

(7) $$T_{⊙}(g)=\frac{1}{\sqrt{d}}(R_{f}f)⊙(R_{\hat{y}}\hat{y})$$

where the dimension of the random multi-threading $d\leq d_{f}\times d_{y}$, $R_{f}$ and $R_{y}$ are random matrices sampled from the Gaussian distribution, and they remain fixed during the training process. The symbol ⊙ represents element-wise multiplication. Compared to the multi-thread mapping T⊗(g), the mapping T⊙(g) can reduce the input data dimension, thus avoiding the issue of dimension explosion.

#### 3.2.4. Dynamic Weight Factor

(a) Information entropy weight *ω*_1_: The information entropy weight factor *ω*_1_ is used in the model to dynamically adjust the training weights of the classifier and discriminator. It is introduced to handle cases where certain training data may have high classification error due to noise or other factors. These data can negatively impact the alignment of classes in the domain discriminator and result in suboptimal transfer performance. The information entropy H(f) is calculated as the sum of the negative log likelihood of the classifier’s predicted probabilities for each class., i.e.,

(8) $$H(f)=\sum_{c=1}^{C}{\hat{y}}_{c}\log{\hat{y}}_{c}$$

where *C* represents the number of classes, and ${\hat{y}}_{c}$ denotes the output value of the softmax function in the classifier corresponding to the unit of the specific class.

The value of *ω*_1_ is determined based on this entropy measure and is used to adjust the importance of the classifier’s training during the domain adaptation process. By incorporating this dynamic weight factor, the model can achieve better adaptation performance and handle uncertain or noisy data more effectively. The class-domain weight factor can be expressed as

(9) $$\omega_{1}=1+e^{-H(f)}$$

Equations (8) and (9) demonstrate that the more complex the input sample, the greater the value of H(f), which consequently results in a smaller value for *ω*_1_. The magnitude of *ω*_1_ dynamically adjusts in response to changes in the input data.

(b) Cross-entropy weight *ω*_2_: In this part, the cross-entropy weight *ω*_2_ is introduced to dynamically balance the relative importance of domain alignment and class alignment. When the feature marginal distributions between domains are far apart, domain alignment is prioritized to reduce the marginal distribution distance. On the other hand, when the feature marginal distributions are close, class alignment is prioritized to reduce the conditional distribution distance. By dynamically adjusting the weight, the convergence speed and class alignment performance of the model can be improved. The specific method is as follows.

First, the A-distance between the inter-domain marginal distribution and the conditional distribution is computed separately as

(10) $$d_{D1}=2(1-2(R_{d1}))$$

(11) $$d_{D2}=2(1-2(R_{d2}))$$

Then, the cross-entropy weight *ω*_2_ is defined as

(12) $$\omega_{2}=\frac{d_{D2}}{d_{D1}+d_{D2}}$$

where *d_D_*_1_ and *d_D_*_2_ are, respectively delineated in Equations (10) and (11). Equation (12) elucidates that, during the model optimization process, the parameter *ω*_2_ is capable of autonomously adjusting in response to the variations in the loss of *D*1 and *D*2.

#### 3.2.5. Strategy

Based on the above derivation, the final optimization objective of DCAA can be summarized as follows:

(13) $$\min_{G}=R_{c}-\lambda \omega_{1}((1-\omega_{2})R_{d1}+\omega_{2}R_{d2})$$

(14) $$\min_{D_{1},D_{2}}=\omega_{1}((1-\omega_{2})R_{d1}+\omega_{2}R_{d2})$$

During the adversarial training process, the following occurs.

Firstly, the parameters of *D*1 and *D*2 are frozen, followed by the optimization of the parameters of *F* and *C* as per Equation (13). As indicated by Equation (13), this procedure aims to decrease classification loss while increasing domain discrimination loss, thereby extracting discriminable domain-invariant features. Additionally, according to Equation (9), the more complex the input data, the smaller the value of *ω*_1_ becomes, thus minimizing the interference of complex data in domain adaptation. Furthermore, as per Equation (12), *ω*_1_ adaptively shifts focus to the domain discriminator with greater loss by computing the losses of the *D*1 and *D*2. 

Subsequently, the parameters of *F* and *C* are frozen, and the parameters of *D*1 and *D*2 are optimized in accordance with Equation (14). This stage of the process is designed to enhance the discriminative abilities of *D*1 and *D*2.

Through the iterative minimax game between them, the feature extraction capability of *F* is continuously enhanced.

### 3.3. Optimization Algorithm

The proposed DCAA algorithm adopts the stochastic gradient descent with backpropagation for training. Let *θ_f_*, *θ_g_*, *θ_d_*_1_ and *θ_d_*_2_ be the parameters of the feature extractor, classifier, domain discriminator, and class discriminator, respectively. α denotes the learning rate.

During the training of the model using backpropagation, the parameters of each part are updated as follows:

(15) $$\theta_{f}=\theta_{f}-\alpha \frac{\partial (R_{c}-\lambda \omega_{1}((1-\omega_{2})R_{d1}+\omega_{2}R_{d2}))}{\partial \theta_{f}}$$

(16) $$\theta_{g}=\theta_{g}-\alpha \frac{\partial (R_{c})}{\partial \theta_{g}}$$

(17) $$\theta_{d1}=\theta_{d1}-\alpha \frac{\partial (R_{d1})}{\partial \theta_{d1}}$$

(18) $$\theta_{d2}=\theta_{d2}-\alpha \frac{\partial (R_{d2})}{\partial \theta_{d2}}$$

The optimization objective of maximizing the domain discriminator loss is achieved by introducing gradient reversal layers between the feature extractor and the domain discriminator, as well as the conditional domain discriminator. The gradient reversal layer multiplies the gradients received from the two domain classifiers by −*λ*. In summary, the training process of CDAA can be delineated as Algorithm 1.
**Algorithm 1: Training process of CDAA**#pre-train feature extractor *F* and classifier *G*Input: source domain data and labels, *F*, *G*for i in [0, epoch1] do: The classification loss is computed using Equation (4). Model parameters are updated utilizing Equation (16). endReturn: pre-trained *F*, *G.*#pre-train Domain Discriminator *D*1 and Conditional Domain Discriminator *D*2.Input: source domain data, target domain data, *D*1, *D*2.for i in [0, epoch1] do: The domain losses for *D*1 and *D*2 are computed using Equations (5) and (6), respectively. Parameters of *D*1 and *D*2 are updated using Equations (16) and (17), respectively.endReturn: pre-trained *D*1, *D*2.# Adversarial trainingInput: source domain data and labels, target domain data, pre-trained F, G, *D*1 and *D*2.for i in [0, epoch2] do: Parameters are optimized using Equation (15) to achieve the optimization objective outlined in Equation (13). Parameters are optimized using Equations (17) and (18) to achieve the optimization objective outlined in Equation (14).endOutput: fault diagnosis model.where ‘epoch1′ and ‘epoch2′, respectively, denote the epochs for pre-training and training.


## 4. Experiments

We used the Case Western Reserve University bearing dataset and the Southeast University gearbox dataset to validate the performance of the method proposed in this paper. The performance is compared with four advanced transfer learning models: DANN [33], CDAN [34], CORAL [35], DATN [36].

The comparison is based on classification accuracy and the model’s stability in various transfer tasks. By conducting these experiments, the effectiveness and applicability of the proposed method in wind turbine systems are validated.

### 4.1. Dataset Description

**Figure 3:**
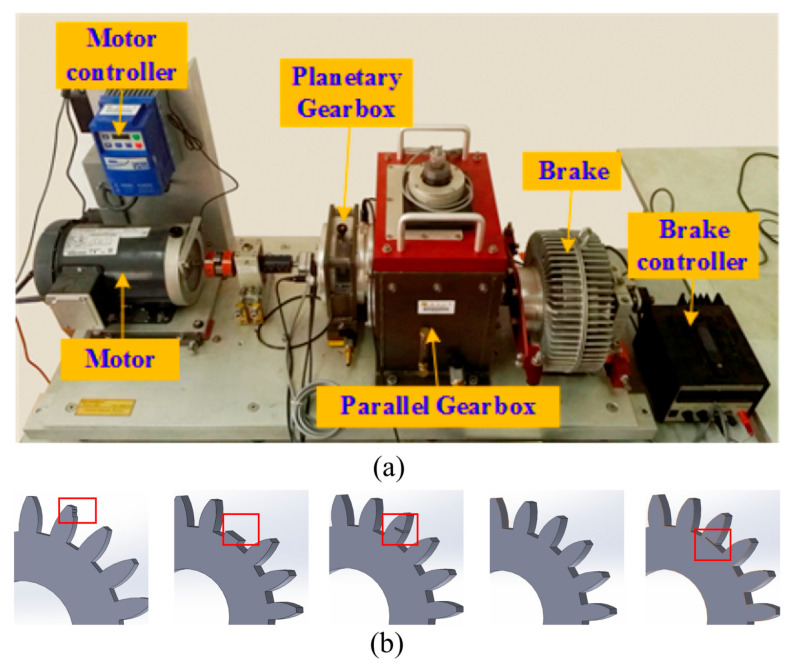
The Southeast University gearbox fault test rig. (**a**) displays the structure of the gearbox test rig, (**b**) shows five different types of gear faults.

Experiment 1: The data were collected by Southeast University from a transmission system dynamic simulator [37]. The experimental setup consists of a driving motor, a planetary gearbox, a parallel-axis gearbox, and a brake. This experimental setup closely resembles the transmission system of a wind turbine, making it suitable for simulating experiments in the context of wind turbine transmission systems. The vibration data of the planetary gearbox were collected using an accelerometer. The dataset includes five different health conditions, namely chipped, miss, root, surface, and healthy, as well as two different operating conditions with the speed–load (rpm-V) conditions, respectively, set at 20-0 and 30-2. The gear fault types are illustrated in Figure 3b. The data were sampled at a frequency of 10,000 Hz, and each category contains 100,000 data samples. In this experiment, a sliding window sampling method was employed to construct the experimental dataset, with a window size of 1024 points in each sample. The dataset was split into training and testing sets with a ratio of 3:1. Specifically, each condition of the two operating conditions contains 3750 samples in the training set and 1250 samples in the testing set. The continuous wavelet transform was utilized to convert the samples into time–frequency images, which serve as inputs to the model.

**Figure 4:**
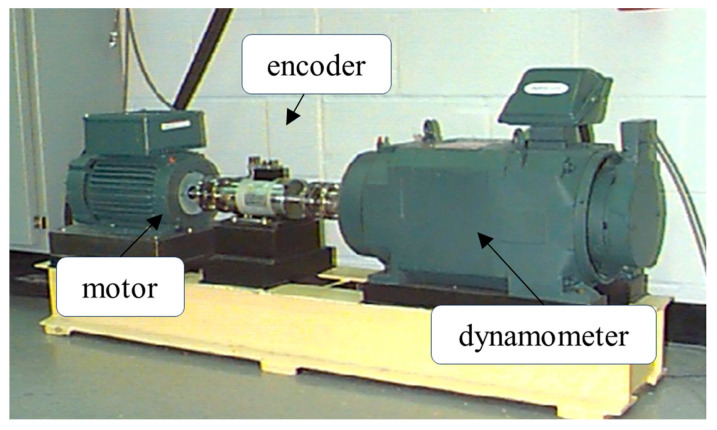
The Case Western Reserve University bearing fault test rig.

Experiment 2: The data for this experiment were obtained from Case Western Reserve University and the experimental setup is shown in Figure 4. The dataset consists of six fault states, namely inner race fault (IR), orthogonal outer race fault at 3 o’clock position (OR@3), orthogonal outer race fault at 12 o’clock position (OR@12), centered outer race fault at 6 o’clock position (OR@6), ball fault (B), and normal state (N). Each fault state has three different levels of damage, with sizes of 0.007 inches, 0.014 inches, and 0.021 inches. The data include four different load(Nm) conditions, labeled as 0, 1, 2, and 3, and were sampled at a frequency of 48 kHz. In this experiment, a sliding window sampling method was used to create the dataset, with each individual data sample containing 1024 points. Each health condition contains 800 points. Similarly, continuous wavelet transform was used to generate time–frequency maps.

### 4.2. Parameter Settings and Results

(1) Experiment 1: The feature extractor is a 2D convolutional neural network with a kernel size of 5 × 5 and a pooling layer size of 2 × 2. A total of 150 epochs were set for training, with an initial learning rate of 0.001. The mini-batch size was set to 128, with 64 samples from the source domain and 64 samples from the target domain. In the training process, only the classifier was trained for the first five epochs. To eliminate the impact of randomness, the results were averaged over 20 experiments. To ensure fairness in the experiments, the same hyperparameters were set for the comparison models, and they were trained for the same number of epochs. The baseline in this study is a convolutional neural network with the same feature extractor, trained only on the source domain data. We evaluate and compare the performance of various models using the accuracy. Accuracy is defined as the ratio of the number of correctly classified samples to the total number of samples in the test set.

The experimental results are shown in Table 2. The baseline, which did not undergo transfer training, achieved an average accuracy of only 67.22% on the target domain. In comparison, DANN, CDAN, CORAL, DATN, and CDAA achieved average accuracies of 80.79%, 82.74%, 71.19%, 87.14%, and 92.56%, respectively.

(2) Experiment 2: The model parameters remain unchanged, and each batch size is set to 64, containing 32 samples from both the source and target domains. During the training process, the classifier is exclusively trained for the first 15 epochs, and the total number of epochs is set to 150. The baseline remains consistent with the previous experiments. There are a total of 12 transfer tasks based on the four operating conditions, and the results are shown in Table 3.

In the baseline convolutional neural network without transfer learning, the average accuracy on the target domain is 67.50%. In the comparative experiments, the proposed CDAA model achieved the highest average accuracy, which is 95.81%. Additionally, the CADA model demonstrated more stable performance across the 12 transfer tasks.

(3) Results Analysis: Indeed, the experimental results clearly demonstrate that while convolutional neural networks (CNNs) have the ability to learn deep features, they struggle with domain shift in transfer tasks, resulting in the lowest accuracy among the tested models.

Among the methods that consider marginal distribution alignment, DANN performs slightly better than CORAL, but their accuracies are both lower than those of the conditional distribution alignment methods. This is because they do not take into account the relationships between sub-domains, leading to suboptimal performance. In the methods that consider conditional distribution alignment, CDAN, DATN, and CDAA perform equally well in some individual tasks, but CDAN and DATN have lower average accuracies across all tasks compared to CDAA. Exactly, CDAA synergizes the alignment of both marginal distributions and conditional distributions, allowing it to perform well in tasks with significant differences between source and target domain data. This adaptability to different data distributions makes CDAA effective in various transfer learning tasks. Taking the two transfer tasks in Experiment 1 as examples, due to the simultaneous variation of rotation speed and load, there is a significant difference in data distribution between the two operating conditions. It is evident that CDAA performs better, achieving a 5.97% improvement compared to DATN. Additionally, the dynamic weighting factors *ω*_1_ and *ω*_2_ in CDAA effectively suppress negative transfer and adaptively adjust the training emphasis on the marginal and conditional distributions based on different data distributions. This capability allows CDAA to adapt to complex and variable operating conditions effectively.

**Figure 5:**
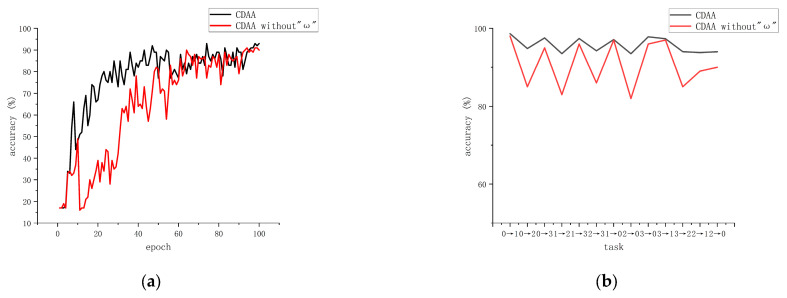
The impact of dynamic weight factors on the model. (**a**) illustrates the impact of dynamic weights on model training. (**b**) demonstrates the effect of dynamic weights on the classification results of the model.

To demonstrate the impact of dynamic weighting factors on the model, we conducted ablation experiments using Experiment 2 as an example. The experimental results are shown in Figure 5. In Figure 5a represents the iteration graph for Task 1 ~ 0, and (b) shows the accuracy of the two methods in each transfer task. It is evident that, during the training process, CDAA with the addition of dynamic weighting factors converges faster and is more stable. Moreover, it performs well in complex transfer tasks and exhibits stronger robustness.

### 4.3. Visualization Analysis

**Figure 6:**
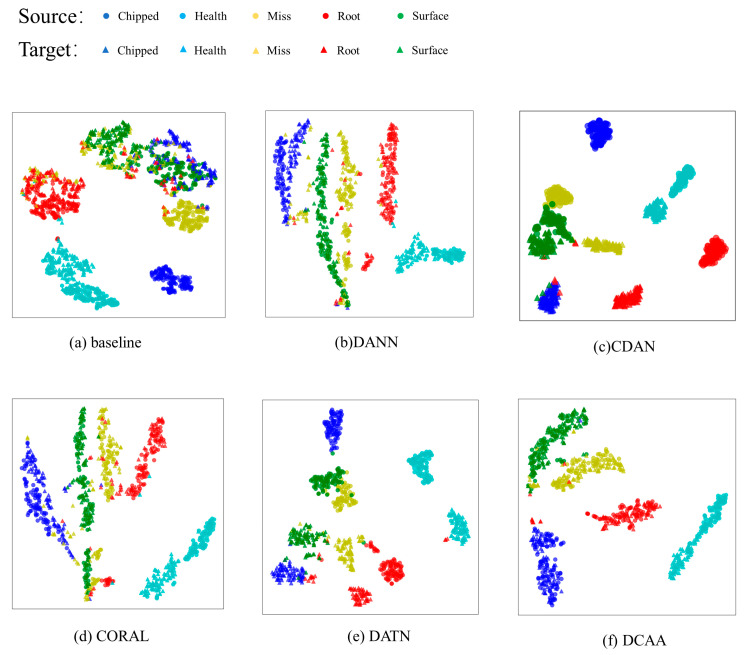
The feature visualization of the 20-0~30-2 task in Experiment 1.

**Figure 7:**
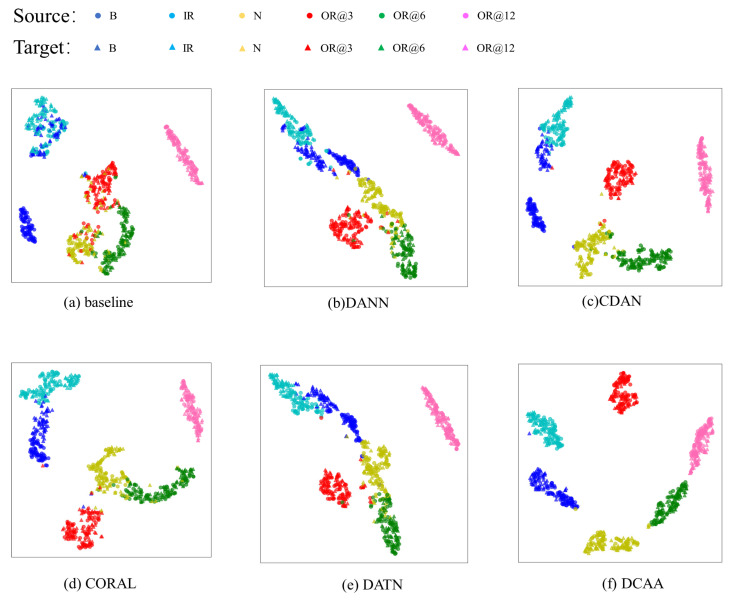
The feature visualization of the 0~1 task in Experiment 2.

To visually demonstrate the effect of DCAA feature distribution alignment, T-SNE is employed for the visualization of feature vectors. We selected the more complex tasks from the two sets of experiments as examples. The results are shown in Figure 6 and Figure 7.

From Figure 6a and Figure 7a, it is evident that in the CNN without the domain adaptation module, the same-class feature distributions from different domains are relatively distant. Furthermore, in the target domain, the overlapping of distributions among some classes hinders effective distinction, resulting in lower accuracy in the target domain. DANN and CORAL are capable of reducing the feature distribution distance between the two domains effectively. However, due to their focus only on aligning the marginal feature distributions, the alignment of some specific class features is not prominent, leading to potential confusion between classes. DATN and CDAA perform conditional distribution alignment for both the source and target domain features, which prevents the feature distance between different fault types from being compressed. As a result, they are more effective in fault classification compared to DANN and CORAL, as they avoid confusion between different classes. CDAA, with the addition of dynamic weight factors, combines both marginal and conditional distribution alignment, effectively reducing the distance between domains and avoiding feature confusion between classes. Figure 6f and Figure 7f clearly demonstrate that DCAA effectively clusters the same-class data from both domains. Moreover, in the target domain, there is strong intra-class compactness and inter-class separability, facilitating the generation of decision boundaries. Therefore, CDAA achieves higher accuracy compared to the first two models.

**Figure 8:**
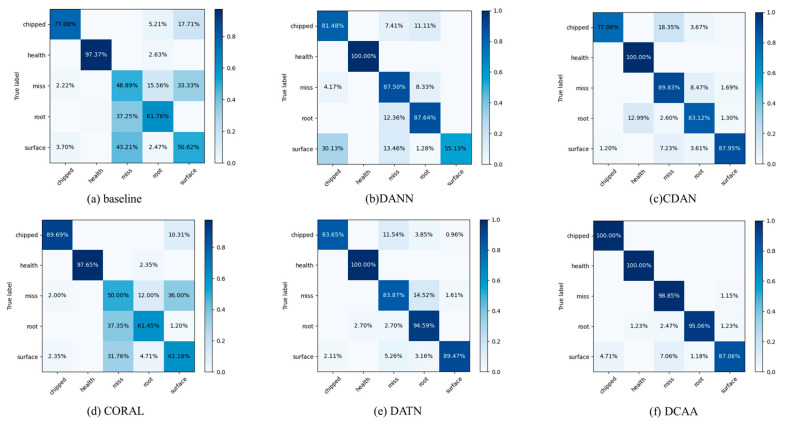
Confusion matrices for various models on the 20-0~30-2 task in Experiment 1.

**Figure 9:**
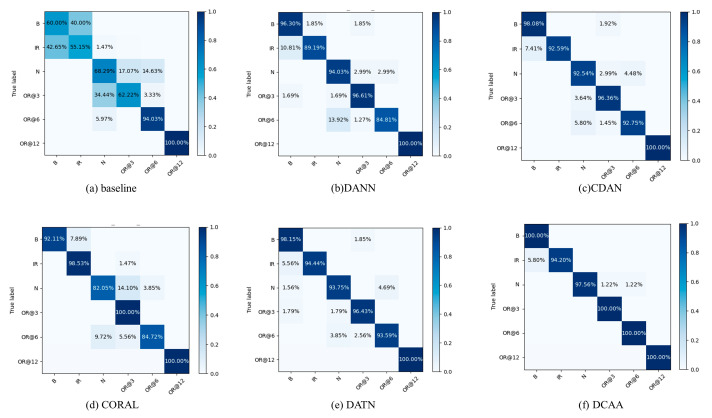
Confusion matrices for various models on the 0~1 task in Experiment 2.

To gain a better understanding of DCAA’s classification performance on each class, we have plotted the confusion matrices for CDAA and other comparative models on the two datasets. Taking the more complex tasks from the two experiments as examples, the confusion matrix results are shown in Figure 8 and Figure 9.

From the confusion matrices, it can be observed that in Experiment 1, the miss, root, and surface fault categories have significant differences in feature distributions. DANN and CORAL, which only perform marginal distribution alignment, struggle to accurately discriminate these three fault categories. The conditional distribution alignment methods can effectively improve this situation, but the accuracy is still influenced by the fault types. CDAA, proposed in this study, adjusts the alignment of both distributions using dynamic weight factors, aiming to minimize the feature distribution distance among the same fault categories. As a result, CDAA performs well in complex fault classification tasks. The same conclusion can be drawn from Experiment 2, demonstrating the robustness of CDAA in different conditions.

## 5. Conclusions

This paper proposes a novel fault diagnosis model for wind turbine gearbox, called CDAA, which can improve fault diagnosis accuracy in the absence of labeled target domain data and reduce the impact of data feature domain distribution under different operating conditions. The model utilizes a convolutional neural network as a feature extractor, taking time-frequency spectrograms generated through wavelet transformation as the input to extract domain-invariant features from vibration signals. Domain discriminator and conditional domain discriminator are employed to measure the marginal distribution distance and class distribution distance between the source and target domain features, respectively. Through adversarial training, the model reduces the feature distribution distance between the two domains. The A-distance is used to evaluate the relative importance of domain alignment and class alignment, and a dynamic weight factor *ω*_2_ is set to balance domain alignment and class alignment training. Moreover, using information entropy as an indicator to measure the data’s classification ability, another dynamic weight factor *ω*_1_ is introduced to balance adversarial training and classification training. Comparative experiments on different datasets demonstrate that the proposed model achieves superior fault diagnosis accuracy compared to other models, achieving accuracies of 92.45% and 95.68%, respectively. The experiments also show that this method is more adaptable to various operating conditions and exhibits stronger applicability in the fault diagnosis of wind turbine gearboxes.

## Figures and Tables

**Table 1:** Feature extractor parameter configuration.

Layer Type	Kernel Size	Stride	Output Size
Input	/	/	(3,128,128)
Conv	(5,5)	1	(6,124,124)
Polling	(2,2)	2	(6,62,62)
Dropout (*p* = 0.5)	/	/	(6,62,62)
Conv	(5,5)	1	(36,58,58)
Polling	(2,2)	2	(36,29,29)
Dropout (*p* = 0.5)	/	/	(36,29,29)
Conv	(3,3)	2	(256,14,14)
Polling	(2,2)	2	(256,7,7)
FC	/	/	(720,1)
Classifier	/	/	(num class, 1)

**Table 2:** Accuracy of various models in experiment 1. Experiment 1 encompasses two transfer tasks under two different operating conditions (the speed–load conditions are, respectively, set at 20 rpm-0 V and 30 rpm-2 V), where ‘a’~’b’ denotes the transfer task with ‘a’ as the source domain and ‘b’ as the target domain.

Transfer Task	Fault Diagnosis Accuracy (%)					
	Baseline	DANN	CDAN	CORAL	DATN	CDAA
20-0~30-2	67.14 ± 1.94	82.68 ± 1.52	84.49 ± 3.66	71.99 ± 6.67	87.32 ± 3.44	**93.66 ± 2.99**
30-2~20-0	66.27 ± 2.97	78.89 ± 1.25	80.78 ± 2.06	70.52 ± 1.61	86.43 ± 1.30	**91.23 ± 2.66**
Average	66.71	80.79	82.64	71.19	86.48	**92.56**

**Table 3:** Accuracy of various models in experiment 2. Experiment 2 encompasses 12 transfer tasks under 4 different operating conditions (the load(Nm) are set at 0, 1, 2, and 3, respectively), where ‘a’~‘b’ denotes the transfer task with ‘a’ as the source domain and ‘b’ as the target domain.

Transfer Task	Fault Diagnosis Accuracy (%)					
	Baseline	DANN	CDAN	CORAL	DATN	CDAA
0~1	73.28 ± 2.79	94.79 ± 1.63	95.71 ± 2.06	92.46 ± 1.61	96.57 ± 1.31	**98.63 ± 2.67**
0~2	65.26 ± 3.01	82.20 ± 1.25	87.92 ± 2.23	82.61 ± 1.62	88.41 ± 1.29	**94.83 ± 1.54**
0~3	71.55 ± 3.11	91.95 ± 1.34	95.46 ± 2.30	89.63 ± 1.96	95.10 ± 1.29	**97.49 ± 2.71**
1~2	63.22 ± 3.94	83.05 ± 1.29	87.92 ± 1.71	81.50 ± 1.30	87.84 ± 2.58	**93.47 ± 1.15**
1~3	72.37 ± 1.91	92.66 ± 1.25	94.46 ± 1.58	92.10 ± 1.71	95.59 ± 1.95	**97.40 ± 1.31**
2~3	62.43 ± 2.86	82.08 ± 2.31	87.77 ± 1.36	80.95 ± 1.40	87.21 ± 2.11	**94.26 ± 2.41**
1~0	73.05 ± 3.12	94.55 ± 1.22	97.01 ± 1.43	91.94 ± 2.31	97.72 ± 1.28	**97.13 ± 1.98**
2~0	62.49 ± 3.44	79.98 ± 1.27	85.23 ± 2.09	81.60 ± 1.66	86.38 ± 2.01	**93.50 ± 2.49**
3~0	70.72 ± 2.95	84.10 ± 1.53	94.53 ± 2.16	86.72 ± 1.42	93.95 ± 1.27	**97.84 ± 2.68**
3~1	70.40 ± 3.51	89.11 ± 1.24	91.06 ± 2.52	91.14 ± 1.81	95.70 ± 1.76	**97.34 ± 2.30**
3~2	60.92 ± 2.99	73.03 ± 1.97	85.69 ± 3.21	70.59 ± 1.89	88.28 ± 1.90	**94.03 ± 1.75**
2~1	64.26 ± 2.79	74.24 ± 1.61	89.27 ± 2.49	72.05 ± 1.84	88.13 ± 2.53	**93.80 ± 2.67**
Average	67.50	85.15	91.00	84.44	91.74	**95.81**

## Data Availability

The data that support the findings of this study are available from the corresponding author upon reasonable request.
